# Molecular phylogeny of Polyneoptera (Insecta) inferred from expanded mitogenomic data

**DOI:** 10.1038/srep36175

**Published:** 2016-10-26

**Authors:** Nan Song, Hu Li, Fan Song, Wanzhi Cai

**Affiliations:** 1 College of Plant Protection, Henan Agricultural University, Zhengzhou, China; 2 Department of Entomology, China Agricultural University, Beijing, China

## Abstract

The Polyneoptera represents one of the earliest insect radiations, comprising the majority of hemimetabolous orders, in which many species have great economic importance. Here, we sequenced eleven mitochondrial genomes of the polyneopteran insects by using high throughput pooled sequencing technology, and presented a phylogenetic reconstruction for this group based on expanded mitochondrial genome data. Our analyses included 189 taxa, of which 139 species represent all the major polyneopteran lineages. Multiple results support the monophyly of Polyneoptera, the monophyly of Dictyoptera, and the monophyly of Orthoptera. Sister taxon relationships Plecoptera + Dermaptera, and Zoraptera + Embioptera are also supported by most analyses. Within Dictyoptera, the Blattodea is consistently retrieved as paraphyly due to the sister group relationship of *Cryptocercus* with Isoptera. In addition, the results demonstrate that model selection, data treatment, and outgroup choice can have significant effects on the reconstructed phylogenetic relationships of Polyneoptera.

Polyneoptera includes some early divergent and diverse hemimetabolous insects, which are constituted by 11 higher taxa conventionally ranked as orders: Mantodea (mantids), Phasmatodea (stick insects), Orthoptera (crickets, katydids, grasshoppers, locusts, etc.), Dermaptera (earwigs), Grylloblattodea (rock crawlers), Zoraptera (zorapterans), Plecoptera (stoneflies), Blattodea (cockroaches), Isoptera (termites), Embioptera (webspinners), and the recently discovered insect order Mantophasmatodea (heelwalkers)^1^,^2^,^3^,^4^. The importance as agriculturally dominant insect pests (e.g. locusts), or natural enemies (e.g. mantids), or vectors of disease-causing pathogens (e.g. cockroaches) has resulted in intense and detailed biological study of many species of Polyneoptera. Phylogenetic relationships within Polyneoptera have been widely researched, and various hypotheses have been raised to explain the evolution of these insects^5^,^6^,^7^,^8^,^9^. However, few of these studies involved a comprehensive taxon sampling, and evaluated systematically the relationships among polyneopteran orders.

The monophyly of the entire Polyneoptera is generally accepted^10^,^11^,^12^, yet phylogenetic relationships within this group are still contentious. The superorder Dictyoptera and the order Orthoptera are two well resolved high level relationships of Polyneoptera. Within the Dictyoptera, the hierarchy arrangement of the clade (Mantodea + (Blattodea + Isoptera)) has been well supported by molecular evidence^12^,^13^,^14^,^15^. Termites are actually social cockroaches, with the family Cryptocercidae as their closest relative rendering the Blattodea paraphyletic, while the Mantodea is the sister group to a monophyletic cockroach/termite clade. Within Orthoptera, two suborders are recognized, namely the Ensifera and Caelifera. The monophyly of Orthoptera is well established by mitochondrial genome data^9^,^16^.

Despite the sister-group relationship between Embioptera and Phasmatodea is well supported by several morphological character systems^17^,^18^,^19^ and molecular studies^12^,^13^,^20^, some analyses alternatively supported a closer relationship between Embioptera and Zoraptera^12^,^21^. Additional hypotheses of embiopteran affinities to other insect lineages had also been suggested^2^. Besides the uncertain placement of Embioptera, a universal consensus on the positions of Zoraptera, Plecoptera, Dermaptera, and Mantophasmatodea remains elusive^15^,^22^,^23^,^24^,^25^,^26^. For example, no less than ten hypotheses on the phylogenetic placement of Zoraptera have been proposed^21^,^27^. As addressed above, both morphological characters of wing base structure^21^ and mitochondrial genome data^11^ supported Zoraptera as sister to Embioptera. However, nuclear genes supported a close relationship between Zoraptera and Dictyoptera^28^,^29^. A more recent study based on genome-scale data recovered a sister-group relation between Zoraptera and Dermaptera^12^. The latter arrangement was also supported by a combined analysis of morphological and molecular data^30^.

Mitogenome has been extensively used to infer phylogeny of insect^3^,^9^,^11^,^16^,^31^,^32^. Compared to the whole genome, mitogenome is more readily sequenced with an array of relatively conserved primers and a reasonable cost. And it contains more phylogenetic information than single or multi-gene data. However, the lineage-specifically elevated rates of nucleotide substitutions or base compositional bias leads to the problem of long-branch attraction/non-stationarity, which lower the phylogenetic resolving power of mitogenome for higher level insect phylogenetics. In model-based phylogenetic analyses, vast majority of evolutionary models assume the stationarity of the replacement rate. That is all taxa in a data set evolving clocklike. The extremely sequence composition heterogeneity may seriously interfere in the stationarity of the data set, and further result in systematic error during inference of phylogeny. Strategies for dealing with these problems have been developed, for example, the advanced evolutionary models accounting for sequence compositional heterogeneity, and data coding methods to avoid the impact of potentially uninformative data partitions.

In the present study, we applied high-throughput, pooled sequencing technology^33^,^34^,^35^ to determine eleven polyneopteran insect mitogenomes (i.e. six from Orthoptera, three from Dermaptera, and each one from Mantodea and Blattodea) to expand currently available mitogenomic data. We conducted a series of molecular phylogenetic analyses to assess the effects of varying analysis parameters including model settings, choice of taxa and data coding methods on phylogenetic inference. Comparisons between different analytical methods were performed, with the aim of providing insights into the suitability of mitogenome for phylogenetic reconstruction of high level relationships within Polyneoptera.

## Materials and Methods

### Taxon sampling

Besides 11 newly sequenced mitogenomes, the remaining mitogenome sequences of each order in Polyneoptera were retrieved from GenBank (Table S1). Two series of data sets were created. Firstly, 154 taxa data set were created, of which ingroup are comprised of 4 species from Dermaptera, 10 from Plecoptera, 18 from Phasmatodea, 10 from Mantodea, 15 from Blattodea, 17 from Isoptera, 18 from Ensifera of Orthoptera, 43 from Caelifera of Orthoptera, and each one from Grylloblattodea, Mantophasmatodea, Zoraptera, and Embioptera. The Archaeognatha (three species), Thysanura (three species), Ephemeroptera (three species), and Odonata (four species) were selected as outgroup. Secondly, to assess the effect of taxa sampling on the phylogenetic inference of Polyneoptera, additional outgroup taxa were added into the data set of 154 taxa to construct 189 taxa data set. The 189 taxa data set included more ougroup species from Paraneoptera (14 species), and Holometabola (21 species).

### DNA extraction and mitogenome assembly

Genomic DNA was extracted from each 95–100% ethanol preserved specimen individually using the TIANamp Micro DNA Kit (TIANGEN BIOTECH CO., LTD) following the manufacturer’s protocol. DNA concentration was measured by Nucleic acid protein analyzer (QUAWELL TECHNOLOGY INC.).

Largely identical quantities of genomic DNA from each of the samples were pooled, and the resulting DNA was quantified to be 1.5 ug. The pooled DNA sample was utilized for library construction and sequenced by the Illumina HiSeq2500 platform. For the sequenced sample, 20 Gb paired-end reads of 125 bp length were generated. The NGSQC-Toolkit v2.3.3^36^ software was used for quality control (avg. Q20 > 85%, avg. Q30 > 80%). Reads containing ambiguous bases (N) or shorter than 88 bp were also removed by NGSQC-Toolkit. Finally, no less than 15 Gb high-quality filtered reads were used in de novo assembly with SOAPdenovo v. 2.0^37^.

To identify the mitogenome assemblies from the pooled sequencing files, three different fragments of mtDNA (*cox1* 5′ region, *cytb* 3′ region and *rrnS* 5′ region) were amplified as “Bait” sequences by standard PCR reactions using primers designed with reference of Simon *et al*.^38^ (primers listed in Table S2). Local BLAST searches were conducted with BioEdit version 7.0.5.3^39^ for each bait sequence reference against all corresponding assemblies. Only hits with 100% pairwise identity were considered a successful identification. The identified mitogenomic sequences were inputted into MITOS web service for initial mitogenome annotation^40^. The resultant gene boundaries were checked and corrected by alignment with published mitogenome sequences of each individual insect order.

### Sequence alignment and data concatenation

All thirty-seven genes of insect mitogenome were used for phylogenetic analyses, namely the 13 protein-coding genes, 22 tRNA genes and two rRNA genes. For protein-coding genes, firstly stop codons were excluded. Subsequently, each was aligned based on the invertebrate mitochondrial genetic code with Perl script TransAlign^41^. Each tRNA of rRNA gene was aligned using MAFFT (version 7)^42^ under iterative refinement method incorporating the most accurate local (E-INS-i) pairwise alignment information. The resulting alignments were checked in MEGA 6^43^, and ambiguously aligned positions were manually excluded prior to phylogenetic analyses. Finally, all alignments were concatenated in a single matrix using FASconCAT_v1.0^44^.

Nucleotide homogeneity across taxa was assessed using the Chi-square test^45^ implemented in PAUP*4.0b10^46^. Potential saturation in the combined protein-coding genes was assessed using the index of substitution saturation (*Iss*)^47^ implemented in the DAMBE 5^48^. The genetic distances between major groups were measured using Kimura 2-parameter, Tamura-Nei, and Maximum Composite Likelihood model in the program MEGA with default settings, based on data set of 189taxa_PCGRNA, and using the Archaeognatha as reference. Nucleotide and amino acid composition were also measured with MEGA.

### Phylogeny

#### Data re-coding and partitioning

To alleviate the effect of substitution saturation of codon positions on phylogenetic estimation, protein-coding genes were re-coded by degenerating all sites including synonymous substitutions to IUPAC ambiguity codes through Degen v1.4 (Degen-code)^49^,^50^. Thus, two concatenated data sets were used in phylogenetic analyses: PCGRNA and PCGDegenRNA. The deduced amino acid sequences from protein-coding genes were also used for tree reconstruction. Totally, five data sets with 154 taxa (i.e. PCG, PCG_AA, PCGDegen, PCGRNA, and PCGDegenRNA) were analyzed using maximum likelihood, and Bayesian inference methods. In addition, the combined data sets of 189taxa_PCGRNA and 189taxa_PCGDegenRNA were used to investigate the effect of taxon sampling on tree building.

Prior to analyses, PartitionFinder^51^ was employed to infer the optimal partitioning strategy. Furthermore, we used the Baysian Information Criterion (BIC) to choose the best model for the final combined nucleotide data set under a greedy search with RAxML^52^. The data blocks were defined by gene types (each of 13 PCGs, each of 22 tRNAs and 2 rRNAs) and by codon position (each of three codon positions for PCGs), totally 63 independent blocks were employed for the full data sets. The partition schemes and best-fit models selected for each data set are provided in Table S3.

#### Tree searches

Maximum likelihood (ML) analyses were conducted using RAxML^52^ as implemented in the CIPRES Portal^53^. Branch support was estimated using rapid nonparametric bootstrapping with 1000 replicates. For concatenated data set, the above determined models were applied to the optimal partitions.

Bayesian analyses were performed using a parallel version (pb_mpi1.5a)^54^,^55^ as implemented on a HP server with twenty-four CPU and 64 G memory. The model CAT-GTR was used for nucleotide analyses, while the model CAT for amino acids. Two chains were run in parallel, and started from a random topology. The Maximum \maxdiff” value to accept was set as 0.1.

The bootstrap supports (BS) of ≥75 and posterior probabilities (PP) of ≥0.95 were considered to be credible support values for tree nodes. All sequence and tree files constructed in this article are available in the TreeBASE (http://purl.org/phylo/treebase/phylows/study/TB2:S19694).

#### Tree topology test

In order to determine whether our mitogenome data significantly reject the alternative tree topologies or hypotheses, the topology test were performed on the data set of 154taxa_PCGDegenRNA. The site-log-likelihood values were calculated under the GTR + I + G model using TREE-PUZZLE (Version 5.2)^56^. And then, the obtained values were used as input for the software CONSEL^57^. All constraint likelihood trees were generated by RAxML^52^ as implemented above.

## Results

### Assembly of mitogenomes

A total of 30 insect species were pooled for high-throughput sequencing. After classification based on morphological and molecular characters, 11 individuals were identified as the polyneopterans and used for this study. The mitogenome sequences for these 11 insects showed a 867–2413 × coverage that generally resulted in contigs of 8,382–16,522 bp (Fig. S1). Except for two species from Dermaptera (Anisolabididae sp.: 8382 bp, and *Labidura japonica*: 10981 bp), the remaining insects have complete or nearly complete mitogenome assemblies, which range from 15,366 bp (*Tridactylus* sp.) to 16,522 bp (*Ducetia* sp.) in length. The missing fragments were mainly located in the control region and the adjacent regions. All newly determined sequences has been deposited in GenBank (accession numbers: KX673195-KX673205).

### Sequence characteristics

Every data set exhibits significant departures from base-compositional homogeneity across taxa (*p* = 0.000, Chi-square test of compositional homogeneity across taxa). Even with Degen-coding treatment, homogeneity could not be retrieved. Thus, non-stationarity was assumed for all data sets in further analyses. The results of the substitution saturation tests showed that the values of substitution saturation index (*Iss*) for the first and second codons in PCGs and all sites of RNAs were significantly smaller than the critical values (*Iss.cSym* or *Iss.cAsym*). However, the *Iss* values of the third codon positions were larger than the *Iss.cAsym* (Table 1). This indicated that the third codon positions might provide poor information for phylogenetics under the assumption of a very asymmetrical true tree.

Pairwise Kimura 2-parameter genetic distance across all main groups ranged from 0.340 (Cryptocercidae) to 0.546 (Strepsiptera). The calculations from both Tamura-Nei and Maximum Composite Likelihood also provided similar results to those based on the Kimura 2-parameter model (Table 2). The results showed that the genetic distances in the mitogenome sequences of Strepsiptera, Zoraptera, Hymenoptera, Embioptera, and Dermaptera were higher than 0.5, while the average genetic distances are lesser than 0.45. The higher genetic distances indicated that the analyzed mitogenome sequences of these insects could have higher evolutionary rate than others.

**Figure 1:**
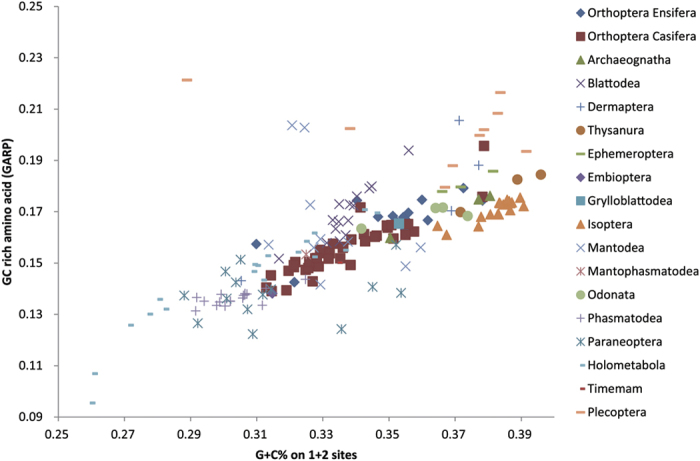
Compositional properties of species analyzed in this study. Both the GC nucleotide content on 1st and 2nd codon positions and the frequency of GC rich amino acids (G, A, R, P) were calculated.

**Figure 2:**
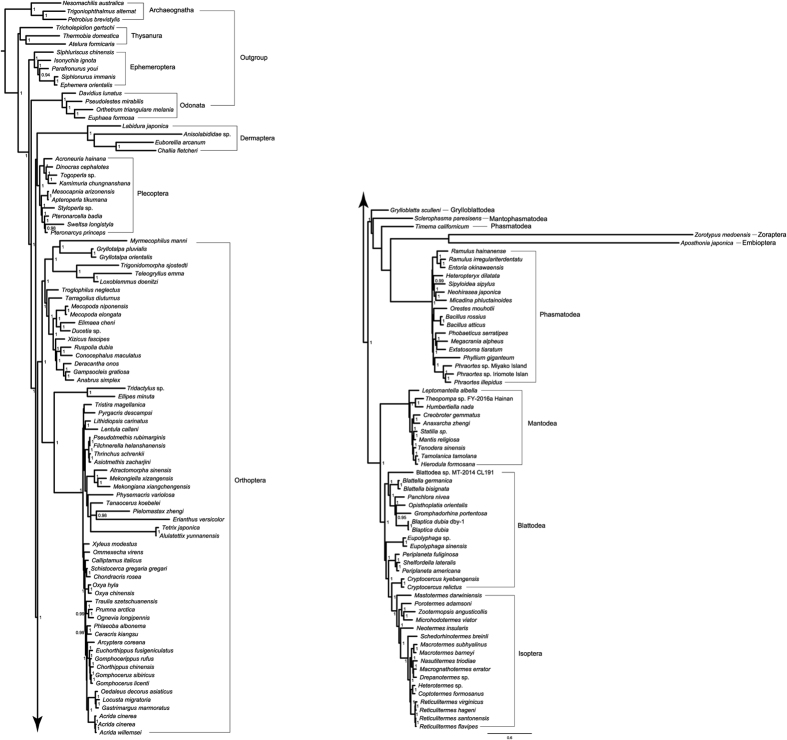
Phylogenetic reconstruction from 154taxa-PCGDegenRNA using software PhyloBayes under CAT-GTR model. Node values represent posterior probabilities (>0.90).

Next, sequence compositional heterogeneity were investigated through calculating the GC nucleotide content on 1st and 2nd sites and the frequency of GC rich amino acids (G, A, R, P) (Fig. 1). The outgroup taxa and some species of both Plecoptera and Isoptera were characterized by high GC% (low AT%), while most of the Holometabola and Paraneoptera had higher AT% (from the mean 65% of Coleorrhyncha to 76% of Hymenoptera). The ingroup polyneopterans had moderate to high GC % and amino acids content of G, A, R and P, and whose values were overlapped with those from outgroup taxa (e.g. the order Archaeognatha and Odonata). These seemed to express a trend on the primitive insect orders with higher GC content in the mtDNA while the relative derived orders with higher AT content. The information shown in the Fig. 2 was consistent with the results of genetic distances presented above, namely that both outgroup Archaeognatha, Thysanura, Odonata, Ephemeroptera and ingroup Polyneoptera) might share similar evolutionary context.

### Maximum-likelihood analysis

The different data treatments under maximum likelihood inference with homogeneous GTR model resulted in different tree topology. Only the data set of 154taxa_PCGDegen produced a monophyletic Polyneoptera, while the nested position of Ephemeroptera rendered Polyneoptera to be non-monophyletic in the trees from the remaining four data sets (i.e. 154taxa_PCG, 154taxa_PCG_AA, 154taxa_PCGRNA, and 154taxa_PCGRNA). In addition, the monophyly of Orthoptera was only recovered by data sets transformed by Degen-coding scheme (i.e. PCGDegenRNA and PCGDegen). Thus, the Degen-coding scheme had a significant effect on the resulting phylogenetic reconstruction on the basis of the current mitogenome data. Besides the monophyly of Polyneoptera and Orthoptera, many other relationships were consistent across data sets and strongly supported regardless of how the data sets were created: the Dermaptera and/or Plecoptera being sister to the remaining taxa within Polyneoptera, the monophyletic Dictyoptera: (Mantodea + (“Blattodea” + (*Cryptocercus* + Isoptera))), the sister-group of (Zoraptera + Embioptera), and the paraphyletic Phasmatodea with respect to the outside position of *Timema*. However, the relationship between Grylloblattodea and Mantophasmatodea was elusive. Only the data set of 154taxa_PCG recovered a sister group of Grylloblattodea and Mantophasmatodea, with weak nodal support (BP = 65). Although the rest analyses presented a close relationship between two insect orders, the Grylloblattodea was retrieved as being relatively primitive to Mantophasmatodea.

### Bayesian analysis

Bayesian analyses under the infinite mixture CAT model, which is devised to account for site-specific amino-acid or nucleotide bias, consistently recovered the monophyly of Polyneoptera (with the exception of the analysis of PCG_AA), and the monophyly of Orthoptera (Fig. 2). The results were in contrast with most of ML analyses, which often supported Polyneoptera and Orthoptera as non-monophyletic. Thus, the site-heterogeneous model has a positive effect on recovering Polyneoptera and Orthoptera. Dermaptera and/or Plecoptera were the earliest branching groups within Polyneoptera. When the data set PCG supported Dermaptera as sister taxon to Plecoptera, the remaining four data sets placed the Dermaptera as the first diverging clade (or as a sister group of outgroup Odonata in the case of the data set of PCG_AA) and the Plecoptera next. The placement of Zoraptera varied between different data treatments. Only the combined data sets (PCGRNA and PCGDegenRNA) supported the sister relationships between Zoraptera and Embioptera, while in the rest analyses from protein-coding genes alone the Zoraptera branched off earlier. The latter arrangement also led Embioptera to have a close affinity to the Phasmatodea (excluding *Timema*). Therefore, it is possible that the addition of RNAs provided the phylogenetic information supporting the sister relationships between Zoraptera and Embioptera. The arrangements of Grylloblattodea, Mantophasmatodea, and Dictyoptera were identical to those from ML analysis.

### Effect of additional outgroup taxa

To assess the effect of the addition of outgroup mitogenome sequence data on the phylogenetic estimation, we reran combined analyses including additional 35 outgroup taxa representatives of the Paraneoptera and Holometabola. The results showed that including more distantly related outgroups resulted in a worse resolution of mitogenomic phylogeny of Polyneoptera. The outgroups Paraneoptera and Holometabola always fall within the ingroup, which rendered the Polyenoptera to be a non-monophyletic group. On the whole, Bayesian inferences on the data sets of 189 taxa performed better than corresponding ML analyses. Specially, using CAT-GTR model effectively dragged the outgroup taxa of Paraneoptera and Holometabola into a more basal position of trees.

### Hypotheses test

Among alternative hypotheses, the following relationships were supported: the monophyletic Polyneoptera, the monophyletic Orthoptera, the sister relationships between Zoraptera + Embioptera, Mantophasmatodea + Grylloblattodea, and Plecoptera + Dermaptera, the superorder Dictyoptera (Mantodea + (“Blattodea” + (*Cryptocercus* + Isoptera))), and the first diverging position of Plecoptera (Table 3). As for the entire Plolyneoptera phylogenies suggested by previous studies of Grimaldi and Engel^58^, Terry and Whiting^30^, Ishiwata *et al*.^15^, Yoshizawa^10^, Ma *et al*.^11^, and Misof *et al*.^12^, the data analyzed in the present study significantly supported the hypothesis in Ma *et al*.^11^, and rejected others.

## Discussion

Although mitochondrial genome was frequently utilized to phylogenetics of insects, some weakness also limited the phylogenetic resolving power of this kind of DNA maker, such as base compositional heterogeneity and lineage-specific evolutionary rate^32^,^59^,^60^. As a result, some previous phylogenetic reconstructions from mitogenome were at odds with the traditional views of insect phylogeny. Strategies to ameliorate these problems usually comprise increased taxonomic sampling, employing different data treatment methods, and implementing better-fitting models^32^,^61^. As an improvement, in this study we applied the strategies as mentioned above and attempted to provide some new insights into the polyneopteran phylogeny.

### Monophyly of Polyneoptera

In previous literatures, some analyses based on nuclear gene sequences found Polyneoptera to be monophyletic^15^,^23^. Whereas, other analyses based on the similar gene fragments using different analytical methods implied a paraphyletic Polyneoptera^13^,^30^. Thus, the uncertainty from the prior studies required additional researches employing more informative molecular makers to reconstruct the phylogeny of this insect group. In this paper, we provided a comprehensive analysis with sample of 37 genes from the whole mitogenomes of 139 species representing 11 polyneopteran orders.

When only one species of Dermaptera (i.e. *Challia fletcheri*) with remarkably long branch was initially included in the phylogenetic reconstruction, it fell within outgroups and emerged as sister taxon to the Ephemeroptera in ML trees (data not shown). This is presumably due to the effect of long-branch attraction. However, increasing three newly sequenced mitogenomes of Dermaptera into the data set successfully broke down this artificial result. Thus, the sequencing of some important and poorly sampled insect groups proved to be beneficial in tree resolution of Polyneoptera.

We also performed topological tests to examine the monophyly of Polyneoptera. All tests supported the hypothesis of the monophyletic Polyneoptera. In particular, when implementing site-heterogeneous model, the bias correlated with homoplasy in the current mitogenome data can be effectively corrected for. The monophyly of Polyneoptera were consistently retrieved across analyses on the basis of differential data treatments.

We also investigate the effect of outgroup choices on ingroup relationships. When including taxa of Paraneoptera and Holometabola into original 154 taxa data sets, the monophyly of Polyneoptera can’t be retrieved by all additional data sets. This may be due to the fact that inclusion of distant outgroups with highly divergent mitogenome sequences led to highly ambiguous alignment, and weakened the resolving power of mitogenome data. This result is in agreement with the limits of mitogenome data in resolving deep phylogeny of insects^61^,^62^. Specially, both Paraneoptera and Holometabola comprised some species with rapid evolutionary mitogenome sequences. Their inclusion further confound the phylogenetic reconstruction of Polyneoptera. The sequence character analyses indicate that most polyneopteran insects share close genetic distance and sequence composition with more primitive insect orders. Thus, selection of the most primitive insects as outgroups for polyneopteran phylogeny estimation is preferred to Paraneoptera and Holometabola.

In short, the hypothesis of polyneopteran monophyly is justified in this study. Furthermore, our analyses demonstrate the applicability of mitogenome data in resolving high level phylogeny of these earliest radiating insect lineages.

### Relationships of the entire orders within Polyneoptera

Among the major orders in Polyneoptera, the Orthoptera are the subject of more published papers than other polyneopterans, due in part to their species richness and economic importance. In this study, PhyloBayes analyses under site-heterogeneous model revealed strong support for the monophyly of Orthoptera (posterior probability = 1.0). By contrast, some analyses under homogeneous GTR model could not reconstruct the monophyletic Orthoptera. In some cases, the suborder Ensifera represented by 18 species split earlier in the tree, while the Caelifera was resolved as a more terminal clade. This led to a non-monophyletic Orthoptera. When investigating the causes underlying this arrangement, we found that the Ensifera have more similar sequence compositional bias to outgroups than the Caelifera (Fig. 1). This factor might potentially result in the deeper position of Ensifera. In the hypothesis tests, the constrained ML tree enforcing a monophyletic Orthoptera was significantly supported, and we were thus able to reject results of the non-monophyletic Orthoptera.

Besides the Orthoptera, another sampling density was centered on the Dictyoptera. The dictyopteran monophyly is generally accepted^22^,^23^,^63^,^64^ and the inter-ordinal relationships of (Mantodea, (“Blattodea”, (*Cryptocercus*, Isoptera))) have received strong support^11^,^12^. In the present study, these relationships among Dictyoptera orders were consistently recovered by all analyses with strong nodal support. Many biological and morphological features suggested a close affinity of *Cryptocercus* and Isoptera (e.g. the homologous sociality between the subsocial *Cryptocercus* and the eusocial termites, and the shared characters of hindgut flagellates of *Cryptocercus* and lower-grade Isoptera). Therefore, our resolution provides a further support for the currently prevailing hypothesis of Dictyoptera^31^,^65^,^66^. Furthermore, every kind of topological test strongly supported this reconstruction.

The Phasmatodea is rendered paraphyletic in all analyses of this study. Because the *Timema* was consistently placed outside the major clade of the Phasmatodea, and led to the paraphyly of Phasmatodea. Within Polyneoptera, the Zoraptera and/ or Embioptera has a closer relation to Phasmatodea. This is congruent with previous morphological and/or molecular analyses^1^,^13^. The study by Ma *et al*.^11^also recovered a sister relationship between Zoraptera and Embioptera, but the long branch attraction artifact were suspected for this relation, due to the very long branches exhibited by both orders. The majority of our analyses recovered this sister relationship, and it received significant support in hypothesis testing. However, the PhyloBayes analyses based on data sets of PCG alone recovered Zoraptera as an earlier diverging lineage, and leaved a sister relation of Embioptera to Phasmatodea. This arrangement is in agreement with result from Misof *et al*.^12^. Thus, more studies need to elucidate the positions of Zoraptera and Embioptera, and the sequencing of more representatives from both insect orders is urgently required.

The Plecoptera is an enigmatic lineage within Polyneoptera, and its phylogenetic placement always changed across different studies. Some authors agreed on the relative primitive position of the Plecoptera^2^,^22^,^23^,^67^, while the sister relationship between Plecoptera and Dermaptera has been suggested by recent molecular studies^15^,^26^. In the PhyloBayes tree from the data set of PCGDegenRNA (Fig. 2), the Plecoptera was recovered as the first lineage to diverge, and formed a sister relationship to Dermaptera. In the tree topology tests, the hypothesis of (Plecoptera + Dermaptera) received most wide support, while the hypothesis of Plecoptera as being a sister group to all other Polyneoptera could not be rejected by the current mitogenome data. Therefore, the placement of Plecoptera remains answered.

As to the Dermaptera, traditional morphology-based analyses have suggested affinities with Dictyoptera, Grylloblattaria, or Embioptera^67^,^68^,^69^, respectively. But none of these arrangements could be strongly upheld^2^. Before this study, only one mitogenome for Dermaptera (i.e. *C. fletcheri*) is available in GenBank. This mitogenome displayed unusual features of strand-bias, and a unique gene arrangement^26^. With the inclusion of three newly sequenced Dermaptera mitogenome sequences, the long branch of *C. fletcheri* is shortened, and the whole Dermaptera consistently appears as the earliest divergent lineage of Polyneoptera.

## Conclusion

In summary, the results obtained in the phylogenetic analyses of the mitogenome data are largely consistent with recently dominant hypotheses of Polyneoptera phylogeny, such as a monophyletic Orthoptera, the Isoptera nested within Blattodea and being as a sister to *Cryptocercus*, and the sister-group relation between Phasmatodea and Embioptera. Conflict in the relationships of Polyneoptera between RAxML and PhyloBayes trees exits mainly on the monophyly of Orthoptera. The result of non-monophyletic Orthoptera generated by RAxML analyses might be ascribed to the sequence compositional bias, which potentially correlates with some systematic errors. Our results also show that the heterogeneous CAT model implemented in the PhyloBayes is a powerful model to account for site-specific nucleotide or amino-acid preferences. By which we can recover a monophyletic Orthoptera and a more reasonable interrelationship of Polyneoptera.

## Additional Information

**How to cite this article**: Song, N. *et al*. Molecular phylogeny of Polyneoptera (Insecta) inferred from expanded mitogenomic data. *Sci. Rep.* **6**, 36175; doi: 10.1038/srep36175 (2016).

**Publisher’s note**: Springer Nature remains neutral with regard to jurisdictional claims in published maps and institutional affiliations.

## Supplementary Material

Supplementary Information

## Figures and Tables

**Table 1:** Saturation testing based on data sets with 154 taxa.

**Gene regions**	**NumOTU**	***Iss***	***Iss.cSym***	**P**	***Iss.cAsym***	**P**
1st codons	32	0.420	0.808	0.0000	0.554	0.0000
2nd codons	32	0.284	0.808	0.0000	0.554	0.0000
3rd codons	32	0.765	0.808	0.0000	0.554	0.0000
all codons	32	0.468	0.818	0.0000	0.572	0.0000
RNAs	32	0.477	0.808	0.0000	0.550	0.0000

Note: two-tailed tests are used.

**Table 2:** Genetic distances for major groups analyzed in this study.

**Jor lineages**	**K2P**	**TN**	**ML**
Cryptocercidae	0.340	0.348	0.347
Blattodea	0.362	0.373	0.372
Mecoptera	0.371	0.383	0.382
Neuroptera	0.371	0.384	0.383
Diptera	0.372	0.384	0.383
Mantophasmatodea	0.394	0.408	0.407
Grylloblattodea	0.398	0.410	0.407
Raphidioptera	0.399	0.414	0.414
Mantodea	0.401	0.415	0.415
Orthoptera	0.404	0.416	0.417
Phasmatodea	0.405	0.420	0.420
Lepidoptera	0.406	0.423	0.420
Psocoptera	0.409	0.425	0.425
Plecoptera	0.412	0.424	0.423
Coleoptera	0.418	0.433	0.434
Odonata	0.419	0.432	0.431
Blattodea	0.423	0.434	0.435
Isoptera	0.432	0.445	0.441
Ephemeroptera	0.434	0.447	0.446
Thysanura	0.462	0.477	0.476
Hemiptera	0.468	0.483	0.487
Dermaptera	0.512	0.534	0.528
Embioptera	0.524	0.547	0.548
Hymenoptera	0.533	0.564	0.563
Zoraptera	0.534	0.555	0.561
Strepsiptera	0.546	0.578	0.577
Avg.	0.429	0.445	0.444

**Table 3:** Tree topology testing based on the data set of 154taxa_PCGDegenRNA.

**Item**	**Hypothesis**	**-Ln likelihood**	**AU**	**KH**	**SH**	**WKH**	**WSH**
1	(Orthoptera)	509138.17	**0.566**	**0.397**	**0.934**	**0.397**	**0.935**
2	(Mantodea, (Blattodea, (*Cryptocercus*, Isoptera))	509152.06	**0.274**	**0.324**	**0.908**	**0.228**	**0.916**
3	(Isoptera, (Blattodea, Mantodea))	509388.38	0.000	0.000	0.004	0.000	0.000
4	(Phasmatodea)	509186.32	0.009	**0.145**	**0.550**	0.006	**0.055**
5	(Phasmatodea, Embioptera)	509245.48	0.016	0.035	**0.156**	0.005	0.042
6	((Timematodea, Mantophasmatodea), Phasmatodea)	509241.00	0.011	0.038	**0.175**	0.007	**0.058**
7	(Zoraptera, Embioptera)	509150.89	**0.532**	**0.332**	**0.915**	**0.332**	**0.979**
8	(Zoraptera, Dermaptera)	509229.22	0.012	0.047	**0.211**	0.006	0.047
9	(Mantophasmatodea, Grylloblattodea)	509151.56	**0.416**	**0.331**	**0.904**	**0.331**	**0.954**
10	(Mantophasmatodea, Mantodea)	509358.74	0.000	0.000	0.005	0.000	0.000
11	(Grylloblattodea, Dermaptera)	509243.56	0.006	0.036	**0.177**	0.003	0.030
12	(Plecoptera, Embioptera)	509338.30	0.002	0.000	0.006	0.000	0.000
13	(Plecoptera, Dermaptera)	509153.77	**0.199**	**0.252**	**0.839**	**0.157**	**0.658**
14	(Plecoptera, all other Polyneoptera)	509141.99	**0.420**	**0.331**	**0.905**	**0.331**	**0.894**
15	(Dermaptera, Embioptera)	509271.56	0.006	0.018	**0.083**	0.001	0.011
16	The monophyletic Polyneoptera	509128.54	**0.655**	**0.603**	**0.978**	**0.603**	**0.981**
17	((Mantodea, (Blattodea, (Cryptocercus, Isoptera))), ((Dermaptera, (Orthoptera, Phasmatodea), (Mantophasmatodea, Grylloblattodea)), (Embioptera, (Plecoptera, Zoraptera)))) (Grimaldi and Engel^58^)	509645.80	0.042	0.000	0.000	0.000	0.000
18	((Orthoptera, (Phasmatodea, Embiidina), ((Mantophasmatodea, Grylloblattodea), (Mantodea, (Blattodea, (Cryptocercus, Isoptera))), (Plecoptera, (Dermaptera, Zoraptera))) (Terry and Whiting^30^)	509413.39	0.000	0.000	0.001	0.000	0.000
19	((Embioptera, Phasmatodea), ((Orthoptera, (Zoraptera, Dictyoptera)), ((Grylloblattodea, Mantophasmatodea), (Dermaptera, Plecoptera)))) (Ishiwata *et al*.^15^)	509579.43	0.002	0.000	0.000	0.000	0.000
20	((Zoraptera, Embioptera), (Plecoptera, ((Orthoptera, Phasmatodea), ((Mantodea, (Blattodea, (Cryptocercus, Isoptera))), ((Grylloblattodea, Mantophasmatodea), Dermaptera))))) (Yoshizawa^10^)	509516.25	0.000	0.000	0.000	0.000	0.000
21	((Plecoptera, Dermaptera), (Orthoptera, ((Mantodea, (Blattodea, (Cryptocercus, Isoptera))), (Grylloblattodea, (((Zoraptera, Embioptera), Phasmatodea), (Timematodea, Mantophasmatodea)))))) (Ma *et al*.^11^)	509146.00	**0.357**	**0.293**	**0.875**	**0.293**	**0.862**
22	((Zoraptera, Dermaptera), (Plecoptera, (Orthoptera, (((Grylloblattodea, Mantophasmatodea), (Phasmatodea, Embioptera)), (Mantodea, (Blattodea, (Cryptocercus, Isoptera))))))) (Misof *et al*.^12^)	509277.26	0.001	0.000	**0.056**	0.000	0.000

Bold indicates the values of p > 0.05.
