# Supplementary material for: Molecular phylogeny of Polyneoptera (Insecta) inferred from expanded mitogenomic data
Source: Sci Rep. 2016 Oct 26;6:36175. doi: 10.1038/srep36175 (PMC5080581; doi:10.1038/srep36175)
Supplement: Supplementary Information [file srep36175-s1.pdf]

Molecular phylogeny of Polyneoptera (Insecta) inferred from expanded mitogenomic data

Nan Song[1 *], Hu Li[2], Fan Song[2], Wanzhi Cai[2 *]

1 College of Plant Protection, Henan Agricultural University, Zhengzhou, China,

2 Department of Entomology, China Agricultural University, Beijing, China

* E-mail: songnan@henau.edu.cn; caiwz@cau.edu.cn

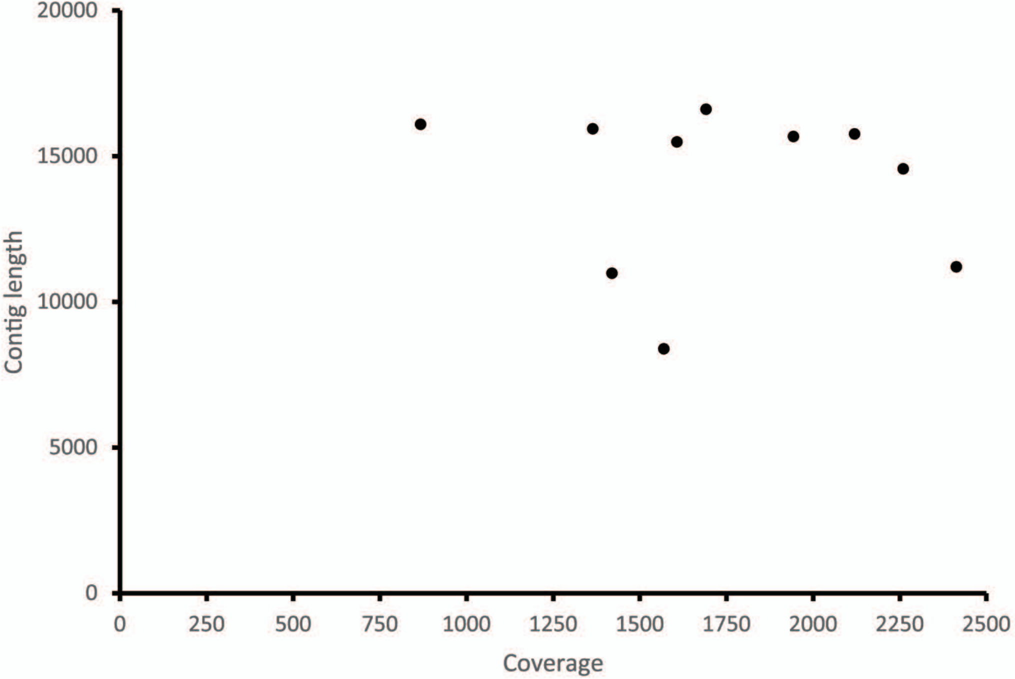

Fig. S1. Mean sequencing coverage versus assembly (contig) length for 11 mitochondrial genomes sequenced in this study.

Table S1. Taxonomic information and GenBank accession numbers for the taxa included in this study.

Item	Subclass	Infraclass	Higher taxon	Order	Superfamily	Family	Species	GenBank accession
	Monocondylia	-	-	Archaeognatha	-	Meinertellidae	*Nesomachilis australica*	NC_006895
	Monocondylia	-	-	Archaeognatha	-	Machilidae	*Petrobius brevistylis*	NC_007688
	Monocondylia	-	-	Archaeognatha	-	Machilidae	*Trigoniophthalmus alternatus*	NC_010532
	Dicondylia	-	-	Thysanura	-	Nicoletiidae	*Atelura formicaria*	NC_011197
	Dicondylia	-	-	Thysanura	-	Lepismatidae	*Thermobia domestica*	NC_006080
	Dicondylia	-	-	Thysanura	-	Lepidotrichidae	*Tricholepidion gertschi*	NC_005437
	Dicondylia	Paleoptera	-	Ephemeroptera	-	Ephemeridae	*Ephemera orientalis*	NC_012645
	Dicondylia	Paleoptera	-	Ephemeroptera	-	Heptageniidae	*Parafronurus youi*	NC_011359
	Dicondylia	Paleoptera	-	Ephemeroptera	-	Siphlonuridae	*Siphlonurus immanis*	NC_013822
	Dicondylia	Paleoptera	-	Ephemeroptera	-	Isonychiidae	*Isonychia ignota*	HM143892
	Dicondylia	Paleoptera	-	Ephemeroptera	-	Siphlonuridae	*Siphluriscus chinensis*	HQ875717
	Dicondylia	Paleoptera	-	Odonata	-	Gomphidae	*Davidius lunatus*	NC_012644
	Dicondylia	Paleoptera	-	Odonata	-	Euphaeidae	*Euphaea formosa*	NC_014493
Outgroup	Dicondylia	Paleoptera	-	Odonata	-	Pseudolestidae	*Pseudolestes mirabilis*	NC_020636
	Dicondylia	Paleoptera	-	Odonata	-	Libellulidae	*Orthetrum triangulare melania*	AB126005
	Dicondylia	Neoptera	Paraneoptera	Cicadomorpha	Membracoidea	Cicadellidae	*Homalodisca coagulata*	AY875213
	Dicondylia	Neoptera	Paraneoptera	Cicadomorpha	Cercopoidea	Aphrophoridae	*Philaenus spumarius*	AY630340
	Dicondylia	Neoptera	Paraneoptera	Coleorrhyncha	-	Peloridiidae	*Hackeriella veitchi*	GQ884145
	Dicondylia	Neoptera	Paraneoptera	Coleorrhyncha	-	Peloridiidae	*Xenophyes cascus*	JF323862
	Dicondylia	Neoptera	Paraneoptera	Fulgoromorpha	Fulgoroidea	Flatidae	*Geisha distinctissima*	FJ230961
	Dicondylia	Neoptera	Paraneoptera	Fulgoromorpha	-	Fulgoridae	*Lycorma delicatula*	EU909203
	Dicondylia	Neoptera	Paraneoptera	Fulgoromorpha	-	Fulgoridae	*Pyrops candelaria*	FJ006724
	Dicondylia	Neoptera	Paraneoptera	Heteroptera	Pentatomoidea	Plataspidae	*Coptosoma bifaria*	EU427334
	Dicondylia	Neoptera	Paraneoptera	Heteroptera	Lygaeoidea	Lygaeidae	*Geocoris pallidipennis*	EU427336
	Dicondylia	Neoptera	Paraneoptera	Heteroptera	Gerroidea	Gerridae	*Aquarius paludum*	FJ456944
	Dicondylia	Neoptera	Paraneoptera	Heteroptera	Pentatomoidea	Pentatomidae	*Halyomorpha halys*	FJ685650
	Dicondylia	Neoptera	Paraneoptera	Heteroptera	-	Anthocoridae	*Orius niger*	EU427341

	Dicondylia	Neoptera	Paraneoptera	Heteroptera	-	Reduviidae	*Triatoma dimidiata*	NC_002609
	Dicondylia	Neoptera	Paraneoptera	Psocoptera	-	Lepidopsocidae	*Lepidopsocid* sp.	NC_004816
	Dicondylia	Neoptera	Holometabola	Coleoptera	-	Histeridae	*Euspilotus scissus*	GU176344
	Dicondylia	Neoptera	Holometabola	Coleoptera	Chrysomeloidea	Cerambycidae	*Monochamus alternatus*	JX987292
	Dicondylia	Neoptera	Holometabola	Coleoptera	-	Curculionidae	*Naupactus xanthographus*	GU176345
	Dicondylia	Neoptera	Holometabola	Diptera	Muscoidea	Muscidae	*Musca domestica*	EU154477
	Dicondylia	Neoptera	Holometabola	Diptera	Trichoceroidea	Trichoceridae	*Paracladura trichoptera*	JN861751
	Dicondylia	Neoptera	Holometabola	Diptera	Oestroidea	Calliphoridae	*Pollenia rudis*	JX913761
	Dicondylia	Neoptera	Holometabola	Hymenoptera	Apoidea	Apidae	*Apis cerana*	GQ162109
	Dicondylia	Neoptera	Holometabola	Hymenoptera	Sphecoidea	Crabronidae	*Philanthus triangulum*	NC_017007
	Dicondylia	Neoptera	Holometabola	Hymenoptera	Vespoidea	Formicidae	*Pristomyrmex punctatus*	AB556946
	Dicondylia	Neoptera	Holometabola	Lepidoptera	Hesperioidea	Hesperiidae	*Ctenoptilum vasava*	JF713818
	Dicondylia	Neoptera	Holometabola	Lepidoptera	Noctuoidea	Lymantriidae	*Gynaephora menyuanensis*	KC185412
	Dicondylia	Neoptera	Holometabola	Lepidoptera	Noctuoidea	Noctuidae	*Spodoptera exigua*	JX316220
	Dicondylia	Neoptera	Holometabola	Mecoptera	-	Bittacidae	*Bittacus pilicornis*	HQ696578
	Dicondylia	Neoptera	Holometabola	Mecoptera	-	Nannochoristidae	*Microchorista philpotti*	HQ696580
	Dicondylia	Neoptera	Holometabola	Mecoptera	-	Panorpidae	*Neopanorpa pulchra*	FJ169955
	Dicondylia	Neoptera	Holometabola	Neuroptera	-	Chrysopidae	*Apochrysa matsumurae*	NC_015095
	Dicondylia	Neoptera	Holometabola	Neuroptera	-	Chrysopidae	*Chrysoperla nipponensis*	NC_015093
	Dicondylia	Neoptera	Holometabola	Neuroptera	-	Ascalaphidae	*Ascalohybris subjacens*	KC758703
	Dicondylia	Neoptera	Holometabola	Raphidioptera	-	Raphidiidae	*Mongoloraphidia harmandi*	NC_013251
	Dicondylia	Neoptera	Holometabola	Strepsiptera	-	Mengenillidae	*Mengenilla moldrzyki*	NC_018545
	Dicondylia	Neoptera	Holometabola	Strepsiptera	-	Stylopidae	*Xenos vesparum*	DQ364229
	Dicondylia	Neoptera	Polyneoptera	Plecoptera	Perloidea	Pteronarcyidae	*Pteronarcys princeps*	NC_006133
	Dicondylia	Neoptera	Polyneoptera	Plecoptera	Perloidea	Perlidae	*Acroneuria hainana*	NC_026104
	Dicondylia	Neoptera	Polyneoptera	Plecoptera	Perloidea	Pteronarcyidae	*Pteronarcella badia*	NC_029248
Ingroup	Dicondylia	Neoptera	Polyneoptera	Plecoptera	Perloidea	Chloroperlidae	*Sweltsa longistyla*	KM216826
	Dicondylia	Neoptera	Polyneoptera	Plecoptera	Perloidea	Perlidae	*Kamimuria chungnanshana*	NC_028076
	Dicondylia	Neoptera	Polyneoptera	Plecoptera	Perloidea	Perlidae	*Togoperla sp. KW-2014*	KM409708

Dicondylia	Neoptera	Polyneoptera	Plecoptera	Perloidea	Perlidae	*Dinocras cephalotes*	NC_022843
Dicondylia	Neoptera	Polyneoptera	Plecoptera	Perloidea	Styloperlidae	*Styloperla sp.*	KR08897
Dicondylia	Neoptera	Polyneoptera	Plecoptera	Nemouroidea	Capniidae	*Apteroperla tikumana*	NC_027698
Dicondylia	Neoptera	Polyneoptera	Plecoptera	Nemouroidea	Capniidae	*Mesocapnia arizonensis*	KP642637
Dicondylia	Neoptera	Polyneoptera	Dermaptera	Pygidicranoidea	Pygidicranidae	*Challia fletcheri*	NC_018538
Dicondylia	Neoptera	Polyneoptera	Dermaptera	Anisolabidoidea	Anisolabididae	***Euborellia arcanum***	**KX673196**
Dicondylia	Neoptera	Polyneoptera	Dermaptera	Anisolabidoidea	Labiduridae	***Labidura japonica***	**KX673201**
Dicondylia	Neoptera	Polyneoptera	Dermaptera	Anisolabidoidea	Anisolabididae	**Anisolabididae sp.**	**KX673197**
Dicondylia	Neoptera	Polyneoptera	Embioptera	-	Oligotomidae	*Aposthonia japonica*	AB639034
Dicondylia	Neoptera	Polyneoptera	Zoraptera	-	Zorotypidae	*Zorotypus medoensis*	NC_026077
Dicondylia	Neoptera	Polyneoptera	Grylloblattodea	-	Grylloblattidae	*Grylloblatta sculleni*	DQ241796
Dicondylia	Neoptera	Polyneoptera	Mantophasmatodea	-	Mantophasmatidae	*Sclerophasma paresisense*	NC_007701
Dicondylia	Neoptera	Polyneoptera	Mantodea	-	Mantidae	*Tamolanica tamolana*	NC_007702
Dicondylia	Neoptera	Polyneoptera	Mantodea	-	Mantidae	***Tenodera sinensis***	**KX673199**
Dicondylia	Neoptera	Polyneoptera	Mantodea	-	Liturgusidae	*Theopompa* sp. FY-2016a Hainan	KU201313
Dicondylia	Neoptera	Polyneoptera	Mantodea	-	Liturgusidae	*Humbertiella nada*	NC_030264
Dicondylia	Neoptera	Polyneoptera	Mantodea	-	Tarachodidae	*Leptomantella albella*	NC_024028
Dicondylia	Neoptera	Polyneoptera	Mantodea	-	Hymenopodidae	*Creobroter gemmatus*	NC_030267
Dicondylia	Neoptera	Polyneoptera	Mantodea	-	Hymenopodidae	*Anaxarcha zhengi*	NC_030268
Dicondylia	Neoptera	Polyneoptera	Mantodea	-	Mantidae	*Statilia* sp.	KU201316
Dicondylia	Neoptera	Polyneoptera	Mantodea	-	Mantidae	*Mantis religiosa*	NC_030265
Dicondylia	Neoptera	Polyneoptera	Mantodea	-	Mantidae	*Tamolanica tamolana*	NC_00770
Dicondylia	Neoptera	Polyneoptera	Mantodea	-	Mantidae	*Hierodula formosana*	NC_029326
Dicondylia	Neoptera	Polyneoptera	Blattodea	Corydioidea	Corydiidae	***Eupolyphaga* sp.**	**KX673200**
Dicondylia	Neoptera	Polyneoptera	Blattodea	Corydioidea	Corydiidae	*Eupolyphaga sinensis*	FJ830540

Dicondylia	Neoptera	Polyneoptera	Blattodea	-	-	Blattodea sp. MT-2014 isolate CL191	KM244690
Dicondylia	Neoptera	Polyneoptera	Blattodea	Blaberoidea	Ectobiidae	*Blattella germanica*	EU854321
Dicondylia	Neoptera	Polyneoptera	Blattodea	Blaberoidea	Ectobiidae	*Blattella bisignata*	JX233805
Dicondylia	Neoptera	Polyneoptera	Blattodea	Blaberoidea	Blaberidae	*Panchlora nivea*	KU684412
Dicondylia	Neoptera	Polyneoptera	Blattodea	Blaberoidea	Blaberidae	*Opisthoplatia orientalis*	NC_029225
Dicondylia	Neoptera	Polyneoptera	Blattodea	Blaberoidea	Blaberidae	*Gromphadorhina portentosa*	NC_030001
Dicondylia	Neoptera	Polyneoptera	Blattodea	Blaberoidea	Blaberidae	*Blaptica dubia* voucher dby-1	NC_029224
Dicondylia	Neoptera	Polyneoptera	Blattodea	Blaberoidea	Blaberidae	*Blaptica dubia*	KU684410
Dicondylia	Neoptera	Polyneoptera	Blattodea	Blaberoidea	Blattidae	*Periplaneta fuliginosa*	AB126004
Dicondylia	Neoptera	Polyneoptera	Blattodea	Blaberoidea	Blattidae	*Shelfordella lateralis*	NC_030003
Dicondylia	Neoptera	Polyneoptera	Blattodea	Blaberoidea	Blattidae	*Periplaneta americana*	NC_016956
Dicondylia	Neoptera	Polyneoptera	Blattodea	Blaberoidea	Cryptocercidae	*Cryptocercus kyebangensis*	NC_030191
Dicondylia	Neoptera	Polyneoptera	Blattodea	Blaberoidea	Cryptocercidae	*Cryptocercus relictus*	JX144941
Dicondylia	Neoptera	Polyneoptera	Isoptera	-	Hodotermitidae	*Microhodotermes viator*	JX144931
Dicondylia	Neoptera	Polyneoptera	Isoptera	-	Kalotermitidae	*Neotermes insularis*	JX144933
Dicondylia	Neoptera	Polyneoptera	Isoptera	-	Mastotermitidae	*Mastotermes darwiniensis*	JX144929
Dicondylia	Neoptera	Polyneoptera	Isoptera	-	Rhinotermitidae	*Coptotermes formosanus*	NC_015800
Dicondylia	Neoptera	Polyneoptera	Isoptera	-	Rhinotermitidae	*Heterotermes* sp.	JX144936
Dicondylia	Neoptera	Polyneoptera	Isoptera	-	Rhinotermitidae	*Reticulitermes flavipes*	EF206314
Dicondylia	Neoptera	Polyneoptera	Isoptera	-	Rhinotermitidae	*Reticulitermes hageni*	EF206320
Dicondylia	Neoptera	Polyneoptera	Isoptera	-	Rhinotermitidae	*Reticulitermes santonensis*	NC_009499
Dicondylia	Neoptera	Polyneoptera	Isoptera	-	Rhinotermitidae	*Reticulitermes virginicus*	EF206318
Dicondylia	Neoptera	Polyneoptera	Isoptera	-	Rhinotermitidae	*Schedorhinotermes breinli*	JX144935
Dicondylia	Neoptera	Polyneoptera	Isoptera	-	Termitidae	*Drepanotermes* sp.	JX144938
Dicondylia	Neoptera	Polyneoptera	Isoptera	-	Termitidae	*Macrognathotermes errator*	JX144939

Dicondylia	Neoptera	Polyneoptera	Isoptera	-	Termitidae	*Macrotermes barneyi*	JX050221
Dicondylia	Neoptera	Polyneoptera	Isoptera	-	Termitidae	*Macrotermes subhyalinus*	JX144937
Dicondylia	Neoptera	Polyneoptera	Isoptera	-	Termitidae	*Nasutitermes triodiae*	JX144940
Dicondylia	Neoptera	Polyneoptera	Isoptera	-	Termopsidae	*Porotermes adamsoni*	JX144930
Dicondylia	Neoptera	Polyneoptera	Isoptera	-	Termopsidae	*Zootermopsis angusticollis*	JX144932
Dicondylia	Neoptera	Polyneoptera	Orthoptera	Acridoidea	Acrididae	*Acrida cinerea*	GU344100
Dicondylia	Neoptera	Polyneoptera	Orthoptera	Acridoidea	Acrididae	*Acrida willemsei*	EU938372
Dicondylia	Neoptera	Polyneoptera	Orthoptera	Acridoidea	Acrididae	*Arcyptera coreana*	GU324311
Dicondylia	Neoptera	Polyneoptera	Orthoptera	Acridoidea	Acrididae	*Calliptamus italicus*	EU938373
Dicondylia	Neoptera	Polyneoptera	Orthoptera	Acridoidea	Acrididae	*Ceracris kiangsu*	GU270284
Dicondylia	Neoptera	Polyneoptera	Orthoptera	Acridoidea	Acrididae	*Chondracris rosea*	GU249619
Dicondylia	Neoptera	Polyneoptera	Orthoptera	Acridoidea	Acrididae	*Chorthippus chinensis*	EU029161
Dicondylia	Neoptera	Polyneoptera	Orthoptera	Acridoidea	Acrididae	*Euchorthippus fusigeniculatus*	HM583652
Dicondylia	Neoptera	Polyneoptera	Orthoptera	Acridoidea	Acrididae	*Gastrimargus marmoratus*	EU513373
Dicondylia	Neoptera	Polyneoptera	Orthoptera	Acridoidea	Acrididae	*Gomphocerippus rufus*	GU294759
Dicondylia	Neoptera	Polyneoptera	Orthoptera	Acridoidea	Acrididae	*Gomphocerus licenti*	GQ180102
Dicondylia	Neoptera	Polyneoptera	Orthoptera	Acridoidea	Acrididae	*Gomphocerus sibiricus*	JX122541
Dicondylia	Neoptera	Polyneoptera	Orthoptera	Acridoidea	Acrididae	*Lithidiopsis carinatus*	JX913770
Dicondylia	Neoptera	Polyneoptera	Orthoptera	Acridoidea	Acrididae	*Locusta migratoria*	X80245
Dicondylia	Neoptera	Polyneoptera	Orthoptera	Acridoidea	Acrididae	*Oedaleus decorus asiaticus*	EU513374
Dicondylia	Neoptera	Polyneoptera	Orthoptera	Acridoidea	Acrididae	*Ognevia longipennis*	EU914848
Dicondylia	Neoptera	Polyneoptera	Orthoptera	Acridoidea	Acrididae	*Oxya chinensis*	EF437157
Dicondylia	Neoptera	Polyneoptera	Orthoptera	Acridoidea	Acrididae	*Phlaeoba albonema*	EU370925
Dicondylia	Neoptera	Polyneoptera	Orthoptera	Acridoidea	Acrididae	*Prumna arctica*	GU294758
Dicondylia	Neoptera	Polyneoptera	Orthoptera	Acridoidea	Acrididae	*Schistocerca gregaria*	GQ491031

Dicondylia	Neoptera	Polyneoptera	Orthoptera	Acridoidea	Acrididae	*Traulia szetschuanensis*	EU914849
Dicondylia	Neoptera	Polyneoptera	Orthoptera	Acridoidea	Acrididae	***Oxya hyla***	**KX673203**
Dicondylia	Neoptera	Polyneoptera	Orthoptera	Acridoidea	Acrididae	***Acrida cinerea***	**KX673195**
Dicondylia	Neoptera	Polyneoptera	Orthoptera	Eumastacoidea	Episactidae	*Pielomastax zhengi*	JF411955
Dicondylia	Neoptera	Polyneoptera	Orthoptera	Grylloidea	Gryllidae	***Trigonidomorpha sjostedti***	**KX673205**
Dicondylia	Neoptera	Polyneoptera	Orthoptera	Grylloidea	Gryllidae	***Loxoblemmus doenitzi***	**KX673202**
Dicondylia	Neoptera	Polyneoptera	Orthoptera	Grylloidea	Gryllidae	*Teleogryllus emma*	EU557269
Dicondylia	Neoptera	Polyneoptera	Orthoptera	Grylloidea	Gryllotalpidae	*Gryllotalpa orientalis*	AY660929
Dicondylia	Neoptera	Polyneoptera	Orthoptera	Grylloidea	Gryllotalpidae	*Gryllotalpa pluvialis*	EU938371
Dicondylia	Neoptera	Polyneoptera	Orthoptera	Grylloidea	Gryllotalpidae	*Myrmecophilus manni*	EU938370
Dicondylia	Neoptera	Polyneoptera	Orthoptera	Acridoidea	Lentulidae	*Lentula callani*	JX913769
Dicondylia	Neoptera	Polyneoptera	Orthoptera	Acridoidea	Ommexechidae	*Ommexecha virens*	JX913775
Dicondylia	Neoptera	Polyneoptera	Orthoptera	Pamphagoidea	Pamphagidae	*Asiotmethis zacharjini*	JX468876
Dicondylia	Neoptera	Polyneoptera	Orthoptera	Pamphagoidea	Pamphagidae	*Filchnerella helanshanensis*	JX468877
Dicondylia	Neoptera	Polyneoptera	Orthoptera	Pamphagoidea	Pamphagidae	*Pseudotmethis rubimarginis*	JX468878
Dicondylia	Neoptera	Polyneoptera	Orthoptera	Pamphagoidea	Pamphagidae	*Thrinchus schrenkii*	GU181288
Dicondylia	Neoptera	Polyneoptera	Orthoptera	Pneumoroidea	Pneumoridae	*Physemacris variolosa*	GU945504
Dicondylia	Neoptera	Polyneoptera	Orthoptera	Pneumoroidea	Pneumoridae	*Tanaocerus koebelei*	JX913774
Dicondylia	Neoptera	Polyneoptera	Orthoptera	Tettigonioidea	Prophalangopsidae	*Tarragoilus diuturnus*	JQ999995
Dicondylia	Neoptera	Polyneoptera	Orthoptera	Acridoidea	Pyrgacrididae	*Pyrgacris descampsi*	JX913771
Dicondylia	Neoptera	Polyneoptera	Orthoptera	Pyrgomorphoidea	Pyrgomorphidae	*Atractomorpha sinensis*	EU263919
Dicondylia	Neoptera	Polyneoptera	Orthoptera	Pyrgomorphoidea	Pyrgomorphidae	*Mekongiana xiangchengensis*	HM583653
Dicondylia	Neoptera	Polyneoptera	Orthoptera	Pyrgomorphoidea	Pyrgomorphidae	*Mekongiella xizangensis*	HM583654
Dicondylia	Neoptera	Polyneoptera	Orthoptera	Rhaphidophoroidea	Rhaphidophoridae	*Troglophilus neglectus*	EU938374
Dicondylia	Neoptera	Polyneoptera	Orthoptera	Acridoidea	Romaleidae	*Xyleus modestus*	GU945503
Dicondylia	Neoptera	Polyneoptera	Orthoptera	Tetrigoidea	Tetrigidae	*Alulatettix yunnanensis*	JQ272702

Dicondylia	Neoptera	Polyneoptera	Orthoptera	Tetrigoidea	Tetrigidae	*Anabrus simplex*	EF373911
Dicondylia	Neoptera	Polyneoptera	Orthoptera	Tetrigoidea	Tetrigidae	*Tetrix japonica*	EU623447
Dicondylia	Neoptera	Polyneoptera	Orthoptera	Tettigonioidea	Tettigoniidae	***Ducetia* sp.**	**KX673198**
Dicondylia	Neoptera	Polyneoptera	Orthoptera	Tettigonioidea	Tettigoniidae	*Conocephalus maculatus*	HQ711931
Dicondylia	Neoptera	Polyneoptera	Orthoptera	Tettigonioidea	Tettigoniidae	*Deracantha onos*	EU137664
Dicondylia	Neoptera	Polyneoptera	Orthoptera	Tettigonioidea	Tettigoniidae	*Elimaea cheni*	GU323362
Dicondylia	Neoptera	Polyneoptera	Orthoptera	Tettigonioidea	Tettigoniidae	*Gampsocleis gratiosa*	EU527333
Dicondylia	Neoptera	Polyneoptera	Orthoptera	Tettigonioidea	Tettigoniidae	*Mecopoda elongata*	JQ917910
Dicondylia	Neoptera	Polyneoptera	Orthoptera	Tettigonioidea	Tettigoniidae	*Mecopoda niponensis*	JQ917909
Dicondylia	Neoptera	Polyneoptera	Orthoptera	Tettigonioidea	Tettigoniidae	*Ruspolia dubia*	EF583824
Dicondylia	Neoptera	Polyneoptera	Orthoptera	Tettigonioidea	Tettigoniidae	*Xizicus fascipes*	JQ326212
Dicondylia	Neoptera	Polyneoptera	Orthoptera	Tridactyloidea	Tridactylidae	***Tridactylus* sp.**	**KX673204**
Dicondylia	Neoptera	Polyneoptera	Orthoptera	Tridactyloidea	Tridactylidae	*Ellipes minuta*	GU945502
Dicondylia	Neoptera	Polyneoptera	Orthoptera	Acridoidea	Tristiridae	*Tristira magellanica*	JX913765
Dicondylia	Neoptera	Polyneoptera	Phasmatodea	Bacilloidea	Bacillidae	*Bacillus atticus*	GU001955
Dicondylia	Neoptera	Polyneoptera	Phasmatodea	Bacilloidea	Bacillidae	*Bacillus rossius*	GU001956
Dicondylia	Neoptera	Polyneoptera	Phasmatodea	-	Diapheromeridae	*Micadina phluctainoides*	AB477466
Dicondylia	Neoptera	Polyneoptera	Phasmatodea	-	Diapheromeridae	*Sipyloidea sipylus*	AB477470
Dicondylia	Neoptera	Polyneoptera	Phasmatodea	Bacilloidea	Heteropterygidae	*Heteropteryx dilatata*	AB477468
Dicondylia	Neoptera	Polyneoptera	Phasmatodea	Bacilloidea	Heteropterygidae	*Orestes mouhotii*	AB477462
Dicondylia	Neoptera	Polyneoptera	Phasmatodea	-	Phasmatidae	*Entoria okinawaensis*	AB477459
Dicondylia	Neoptera	Polyneoptera	Phasmatodea	-	Phasmatidae	*Extatosoma tiaratum*	AB642680
Dicondylia	Neoptera	Polyneoptera	Phasmatodea	-	Phasmatidae	*Megacrania alpheus adan*	AB477471
Dicondylia	Neoptera	Polyneoptera	Phasmatodea	-	Phasmatidae	*Neohirasea japonica*	AB477469
Dicondylia	Neoptera	Polyneoptera	Phasmatodea	-	Phasmatidae	*Phobaeticus serratipes*	NC_014678
Dicondylia	Neoptera	Polyneoptera	Phasmatodea	-	Phasmatidae	*Phraortes illepidus*	NC_014695
Dicondylia	Neoptera	Polyneoptera	Phasmatodea	-	Phasmatidae	*Phraortes* sp. Miyako Island	AB477465
Dicondylia	Neoptera	Polyneoptera	Phasmatodea	-	Phasmatidae	*Phraortes* sp. Iriomote Island	AB477464
Dicondylia	Neoptera	Polyneoptera	Phasmatodea	-	Phasmatidae	*Ramulus hainanense*	FJ156750
Dicondylia	Neoptera	Polyneoptera	Phasmatodea	-	Phasmatidae	*Ramulus irregulariterdentatus*	NC_014702

Dicondylia	Neoptera	Polyneoptera	Phasmatodea	Phyllioidea	Phylliidae	*Phyllium giganteum*	AB477461
Dicondylia	Neoptera	Polyneoptera	Phasmatodea	Timematoidea	Timematidae	*Timema californicum*	DQ241799

Note: Bold indicates the species newly sequenced in this study.

Table S2. Species-specific primers designed for amplifying "Bait" sequences and control region.

Upstream primers	Sequence (5'-3')	Downstream primers	Sequence (5'-3')
cox1_F	TATTGGTGGTTTTGGAAATTG	cox1_R	GCTCGTGTATCAACGTCTATTCC
cytb_F	GTATTACCATGAGGACAAATATC	cytb_R	TTCAACTGGTTGTATCCCAATTCA
rrnS_F	GTACATTTACTTTGTTACGACTT	rrnS_R	GTGCCAGCAGTTGCGGTTAAAC

Note: F, forward; R, reverse.
